# Developmental Profiling of Tropomyosin Expression in Mouse Brain Reveals Tpm4.2 as the Major Post-synaptic Tropomyosin in the Mature Brain

**DOI:** 10.3389/fncel.2017.00421

**Published:** 2017-12-22

**Authors:** Alexandra K. Suchowerska, Sandra Fok, Holly Stefen, Peter W. Gunning, Edna C. Hardeman, John Power, Thomas Fath

**Affiliations:** ^1^Neurodegeneration and Repair Unit, School of Medical Sciences, University of New South Wales, Sydney, NSW, Australia; ^2^Neuron Culture Core Facility, University of New South Wales, Sydney NSW, Australia; ^3^Cellular and Genetic Medicine Unit, School of Medical Sciences, University of New South Wales, Sydney, NSW, Australia; ^4^Translational Neuroscience Facility, School of Medical Sciences, University of New South Wales, Sydney, NSW, Australia

**Keywords:** tropomyosin, postsynaptic compartment, actin cytoskeleton, brain development, synaptosomes

## Abstract

Nerve cell connections, formed in the developing brain of mammals, undergo a well-programmed process of maturation with changes in their molecular composition over time. The major structural element at the post-synaptic specialization is the actin cytoskeleton, which is composed of different populations of functionally distinct actin filaments. Previous studies, using ultrastructural and light imaging techniques have established the presence of different actin filament populations at the post-synaptic site. However, it remains unknown, how these different actin filament populations are defined and how their molecular composition changes over time. In the present study, we have characterized changes in a core component of actin filaments, the tropomyosin (Tpm) family of actin-associated proteins from embryonal stage to the adult stage. Using biochemical fractionation of mouse brain tissue, we identified the tropomyosin Tpm4.2 as the major post-synaptic Tpm. Furthermore, we found age-related differences in the composition of Tpms at the post-synaptic compartment. Our findings will help to guide future studies that aim to define the functional properties of actin filaments at different developmental stages in the mammalian brain.

## Introduction

The actin cytoskeleton plays an integral role in the structural and functional maturation of neurons. At central nervous system synapses, continuous remodeling of actin filaments (F-actin) is the basis for supporting changes required for learning and memory formation. The pre-synaptic actin cytoskeleton is involved in the trafficking and endocytosis of synaptic vesicles. The rich post-synaptic actin network is critical for the delivery and anchorage of various neurotransmitters and other molecules required for synaptic transmission. Within the post-synaptic compartment, the F-actin network can further be differentiated into three filament populations based on their distinct turnover rates (Honkura et al., 2008). The varying roles and organization of the synaptic actin cytoskeleton is largely attributed to the vast array of actin-associated proteins, which modulate its structure, function, motility and turnover. Therefore, the better understanding of the localization and expression profile of key actin-associated proteins at the synapse, will aid in our understanding of the regulation of synaptic F-actin populations.

Tropomyosins (Tpms) are a family of actin associated-proteins, which form a coiled-coil dimer along the major α-helical groove of actin filaments (Phillips et al., 1979). Thus far, over 40 isoforms of Tpms have been identified in mammals, arising from the alternative splicing of four different genes (Tpm1-4) (Geeves et al., 2015). This diversity in Tpm isoforms has been postulated to bestow the actin cytoskeleton with its diversity and has been described as an evolutionary advantage to diversify actin filament composition (Gunning et al., 2005; Gunning et al., 2015a). Due to their localization, Tpms are able to associate with both F-actin and actin-binding proteins, thereby mediating the direct access of actin-binding proteins to F-actin. Tpms also have isoform specific interactions with F-actin and actin-binding proteins. The overexpression of Tpm1.12 results in recruitment and binding of cofilins to F-actin, leading to the formation of shorter, less stiff F-actin (Bryce et al., 2003). Conversely, the overexpression of Tpm3.1 and Tpm4.2 increases cofilin phosphorylation, inactivating cofilin and reducing its binding to actin (Bryce et al., 2003; Curthoys et al., 2014). Recent *in vitro* data, however, indicates that Tpm3.1, Tpm3.2 and Tpm4.2 are not able to efficiently protect F-actin from the severing action of ADF/cofilin (Gateva et al., 2017; Ostrowska et al., 2017). The discrepancy in the interaction of Tpms with ADF/cofilin may be due to the regulation *in vivo* by Tpms of upstream kinases and/or phosphatase of cofilin- an interaction that is absent in reconstituted *in vitro* systems. Therefore, although it is clear that Tpms define distinct filamentous actin populations in different cell populations and subcellular compartments, further work is needed to better understand the discrepancies between *in vivo* and *in vitro* studies.

In neuronal tissue, products from three of the four *Tpm* genes are present (*Tpm1, Tpm3*, and *Tpm4*) (for a detailed review, see Gray et al., 2017). Limited studies have shown the segregation of different Tpm isoforms at the synapse. The Tpm1 products Tpm1.10 and Tpm1.12 have been shown to be specific to the pre-synaptic compartment in both mice and rats (Had et al., 1994; Weinberger et al., 1996; Vrhovski et al., 2003; Guven et al., 2011). By contrast, Tpm4.2 has been shown to localize to the post-synaptic compartment in mouse and rat primary cultured neurons (Had et al., 1994; Guven et al., 2011). Products from *Tpm3* have been shown to localize post-synaptically, however it is still unclear which of the 9 endogenous *Tpm3* isoforms localize to the post-synaptic compartment (Guven et al., 2011). Furthermore, there is a lack of information on age-dependent localization of Tpms in the pre- and post-synaptic compartments.

In this study, we utilized a subcellular fractionation approach to determine whether the pool of Tpms in the post-synaptic compartment changes over time in the brains of mice. We confirmed previous findings indicating the segregation of *Tpm1* products to the pre-synaptic compartment and *Tpm3* and *Tpm4* products to the post-synaptic compartment. Importantly, we found Tpm4.2 to be the major PSD-associated Tpm isoform in older mice.

## Materials and Methods

### Ethics Statement

All procedures were conducted in accordance with the Australian Code of Practice for the Care and Use of Animals for Scientific Purposes, and were approved by the University of New South Wales Animal Care and Ethics Committee.

### Post-synaptic Density Preparation

Animals were euthanized at E16.5 (for total brain homogenate only), 1, 3, and 7 months of age. Entire brains were removed and snap frozen from 4 biological replicates. Brain homogenates were prepared by homogenizing with 6 volumes of buffer A (0.1 mM EDTA, 0.1 mM EGTA, 0.25 mM PMSF and 1 mM HEPES at pH 7.4) containing 0.32 M sucrose. One, 3 and 7-month-old brains were further processed for synaptosomal preparations as previously described (Frohlich et al., 2017). Synaptic plasma membranes were prepared from frozen mouse brains according to procedures of Monahan and Michel (Monahan and Michel, 1987). The homogenate was centrifuged twice at 1,000 *g* for 7 min. An aliquot from the combined supernatants was saved as the brain homogenate fraction and the rest was centrifuged at 38,900 *g* for 45 min. The pellet was resuspended in 2 volumes of 2 mM Tris-acetate at pH 8.0 and applied on top of buffer A containing 1.2 M sucrose. After centrifugation at 230,000 *g* for 45 min, the interface was collected and applied on top of a layer of buffer A containing 0.9 M sucrose. After centrifugation at 230,000 *g* for 45 min, the pellet contains the synaptic plasma membrane fraction. Synaptic junctions (synaptosomes) were isolated according to procedures of Chang et al. (1991). Synaptic plasma membranes were homogenized in two volumes of buffer B (0.2 mM HEPES, 0.05 mM CaCl_2_, pH 7.4) and centrifuged at 65,000 *g* for 20 min, twice. The pellet was then resuspended in one volume buffer B and 2 volumes buffer C (2 M HEPES, pH 7.6 in 0.4% Triton X-100), loaded onto 1 M sucrose solution, and centrifuged at 85,000 *g* for 1 h. An aliquot of the resulting pellet was saved as the synaptosome fraction. The pure postsynaptic density fraction was prepared by the procedures of Cohen et al. (1977). In brief, synaptosomes were diluted to 4 mg of protein/ml by solution D (5 mM CaCl_2_ and 6 mM Tris-HCl at pH 8.1), mixed with an equal volume of solution E [0.32 M sucrose, 1% (vol/vol) Triton X-100 and 12 mM Tris-HCl at pH 8.1] incubated on ice for 15 min and finally centrifuged at 32,800 *g* for 20 min. The resulting pellet was resuspended in solution D and applied on top of a step gradient containing 1.0, 1.5 and 2.0 M sucrose with 1 mM NaHCO_3_ in the 1.0 and 1.5 M fractions. After centrifugation at 200,000 *g* for 2.5 h, the band at the interface between 1.5 and 2.0 M sucrose layers was collected. The sample was then mixed with an equal volume of a solution containing 150 mM KCl and 1% Triton X-100 and centrifuged at 200,000 *g* for 20 min. The resulting pellet was resuspended in 50 μM CaCl_2_ and 6 mM Tris-HCl at pH 8.1 and used as the PSD fraction. All fractions were analyzed by Western-blotting, with confirmation of clean fractionations shown in Supplementary Figure 1.

### Western-Blotting

Protein concentration was determined by DC-Protein assay (Bio-Rad laboratories) and 10 μg of protein was mixed with 4x Laemmli reducing sample buffer, separated by SDS-PAGE, and transferred onto PVDF membranes (Millipore). Ponceau staining was used to control for loading, particularly between samples of different fractions. Membranes were blocked in 5% milk powder or BSA in 0.1% Tween-TBS and probed with the primary antibodies as described in **Table 1** overnight at 4°C. Detailed characterization of these antibodies has previously been described in (Schevzov et al., 2011). After washing with 0.1% Tween in TBS HRP-conjugated secondary antibodies (GE Healthcare, Rydalmere, NSW, Australia) were applied. Membranes were developed using Luminata Crescendo western HRP Solution (Millipore) and imaged using the GelDoc system (BioRad). Blots were then incubated with Actin (Abcam mab 1501) and PSD-95 (Merck Millipore mab1596) to once again control for loading. Quantification of Western blots was performed via densitometry, using Image J (version 1.47). Signal intensity was normalized to total protein levels, as determined by the Ponceau stain. An independent sample size of either 3 or 4 mice was used.

**Table 1:** Antibodies used for the detection of different tropomyosin antibodies via Western blotting.

Antibody name	Peptide sequence/Immunogen	Tropomyosin isoform detected	Dilution	Dilution buffer	Detailed characterization
α/9c (5-54) mouse monoclonal hybridoma supernatant	HQLEQNRRLT NELKLALNED	Tpm1.10 and Tpm1.12	1:500	BSA	Vrhovski et al., 2003; Schevzov et al., 2011
γ/9a mouse monoclonal affinity purified ascites	CDELYAQKLKYKA ISDELDHALNDMTSI	Tpm3.3, Tpm3.5, Tpm3.8 and Tpm3.9	1:250	Skim milk	Schevzov et al., 2011
γ/9c mouse monoclonal affinity purified ascites	CERLYSQLERNRLL SNELKLTLHGL	Tpm3.4 and Tpm3.7	1:250	Skim milk	Schevzov et al., 2011
γ/9c sheep polyclonal affinity purified ascites	CERLYSQLERNRLL SNELKLTLHGL	Tpm3.4 and Tpm3.7	1:250	Skim milk	Schevzov et al., 2011
γ/9d (2G10.2) mouse hybridoma supernatant	DKLKCTKEEHLCTQRMLD QTLLDLNEM	Tpm3.1 and Tpm3.2	1:500	Skim milk	Schevzov et al., 2011
γ/9d sheep polyclonal affinity purified ascites	DKLKCTKEEHLCTQRM LDQTLLDLNEM	Tpm3.1 and Tpm3.2	1:500	Skim milk	Schevzov et al., 2005, 2011
δ9d (WD4/9d) Tm4 rabbit affinity purified ascites	KEENVGLHQTLDTLN	Tpm4.2	1:250	BSA	Hannan et al., 1998; Schevzov et al., 2005, 2011

*Detailed characterization of antibodies used in this study has previously been described by (Schevzov et al., 2011), including lack of cross reactivity of antibodies for other tropomyosin antibodies.*

Quantitation of absolute levels of Tpm isoforms from *Tpm3* and *Tpm4* in the brain homogenate and PSD fractions was performed as previously described (Schevzov et al., 2011; Jalilian et al., 2015). Briefly, known concentrations of His-tagged recombinant Tpm expression constructs (Schevzov et al., 2011) were separated alongside 1-month-old brain homogenate samples on SDS-PAGE gels and subsequently processed for Western blotting. Densitometry was performed as mentioned above, with the amount of Tpm from Tpm3 9a, Tpm3 9c, Tpm3 9d, and Tpm4.2 calculated in the brain homogenate (ng of Tpm protein expressed per μg of total cellular protein). The amount of Tpm in the PSD fractions of 1, 3, and 7-months old mice was calculated using the blots in **Figures 4**–**7**. Given the amount of brain homogenate and PSD protein loaded was 10 μg, and the amount of Tpm protein in the brain homogenate was now known, the ng of Tpm protein expressed per μg of total cellular protein in the PSD fraction was determined.

### Statistical Analysis

Expression levels in brain homogenates and cellular fractionations were analyzed by one-way ANOVA followed by *t*-Test for individual comparisons. Graphs were generated in GraphPad Prism (Version 7.03). All data in graphs are shown as mean ± SEM.

## Results

### Expression of Tpm Isoforms in the Brains of Mice Changes with Age

**FIGURE 1:**
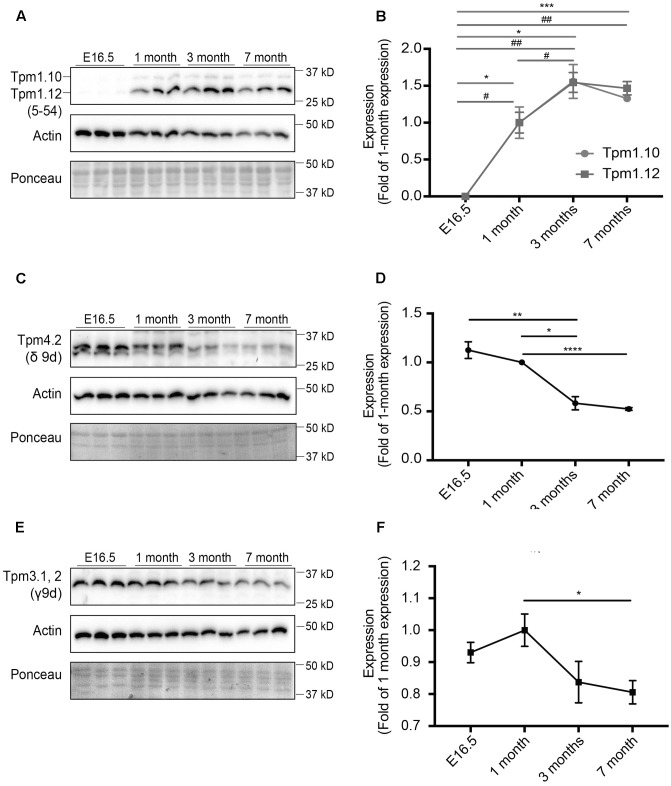
Expression profile of tropomyosin (Tpm) isoforms in the brain throughout development and aging. Western blot analysis and quantification of E16.5, 1, 3, and 7-months-old mouse brain tissue for Tpm expression. **(A)** Tpm1.10 and Tpm1.12 expression, as detected by the 5–54 antibody. **(B)** Quantification of expression of Tpm1.10 and Tpm1.12 expression (^∗^) indicates significance for Tpm1.10, (^#^) indicates significance for Tpm1.12. **(C)** Expression of Tpm4.2 as detected by the δ9d antibody. **(D)** Quantification of **(C)**. **(E)** Expression of Tpm3.1, 2, as detected by the γ9d antibody. **(F)** Quantification of **(E)**. (mean ± SEM, *n* = 3, ^∗/#^*P* < 0.05, ^∗∗/##^*P* < 0.01, ^∗∗∗^*P* < 0.001, ^∗∗∗∗^*P* < 0.0001).

**FIGURE 2:**
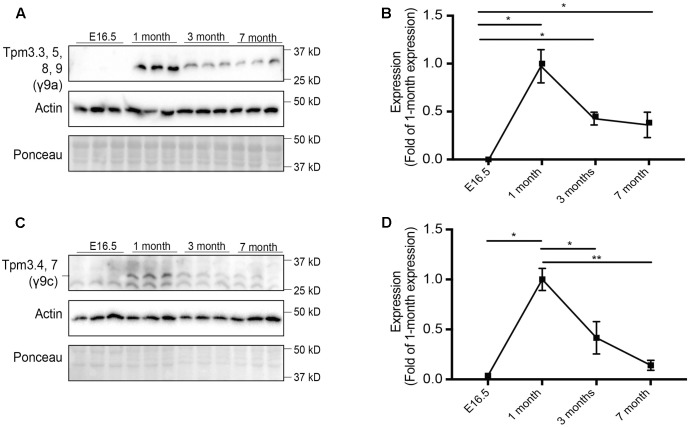
Expression profile of tropomyosin (Tpm) isoforms in the brain throughout development and aging. Western blot analysis and quantification of E16.5, 1, 3, and 7-months old-mouse brain tissue for Tpm expression. **(A)** Tpm3.3, 5, 8, 9 expression as detected by the γ/9a antibody. **(B)** Quantification of **(A)**. **(C)** Expression of Tpm3.4,7, as detected by the γ9c antibody. **(D)** Quantification of **(C)**. (mean ± SEM, *n* = 3, ^∗^*P* < 0.05, ^∗∗^*P* < 0.01).

In this study, we analyzed changes in the expression of different Tpm isoforms in mice of varying ages. Mouse brains from E16.5, 1, 3, and 7 months old mice were analyzed, with expression levels normalized to expression at 1 month of age (**Figures 1**, **2**). Characterization of isoforms from the *Tpm1* gene, utilizing the 5–54 antibody, revealed a similar pattern of expression for both Tpm1.10 and Tpm1.12 (**Figures 1A,B**). Both Tpm1.10 and Tpm1.12 were absent in the brains of E16.5 embryos. Expression of both isoforms was detected from 1 month of age, with a significant increase in expression of Tpm1.12 between 1- and 3-months of age.

Expression of Tpm4.2 was analyzed utilizing the δ9d (WD4/9d) antibody (**Figures 1C,D**). There was an overall reduction in expression of Tpm4.2 with age, particularly by 7 months of age.

Expression of Tpm3.1 and Tpm3.2 in the brains of mice was determined using the γ/9d sheep polyclonal affinity purified ascites antibody (**Figures 1E,F**). Although there was no linear trend in expression observed, by 7 months of age, there was a significant decrease in expression of Tpm3.1/2 compared to expression at 1 month of age.

Expression of Tpm3 exon 9a-containing isoforms (Tpm3.3, Tpm3.5, Tpm3.8, and Tpm3.9) was analyzed using the γ/9a mouse monoclonal affinity purified ascites antibody (**Figures 2A,B**). No expression was detected at E16.5. The highest level of expression was observed at 1 month of age, after which expression decreased.

Expression of the Tpm3 exon 9c-containing isoforms (Tpm3.4 and Tpm3.7) was determined using the γ/9c mouse monoclonal affinity purified ascites antibody (**Figures 2C,D**). A similar pattern of expression as Tpm3 exon 9a-containing isoforms was observed. Minimal expression was detected at E16.5, with a significant increase in expression observed at 1 month of age. Thereafter, a significant decrease in expression levels of Tpm3.4 and Tpm3.7 was observed.

### Both Tpm1.10 and Tpm1.12 Are Present at the Synaptic Junction, but Absent from the Post-synaptic Density Fraction

**FIGURE 3:**
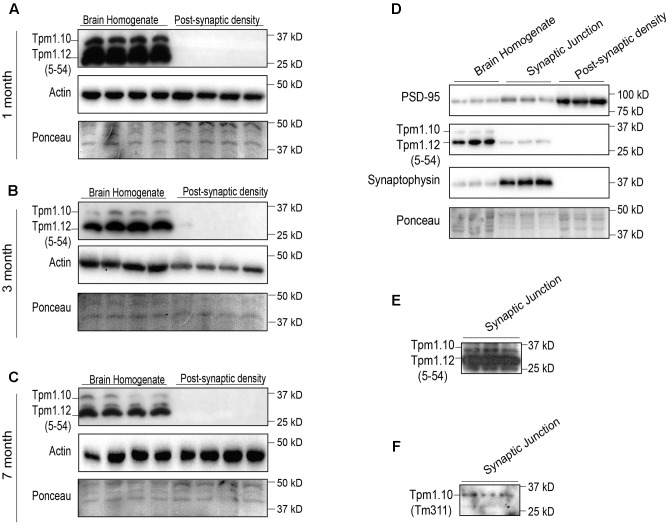
Expression profile of tropomyosin (Tpm) isoforms Tpm1.10 and Tpm1.12 (utilizing the 5–54 and Tm311 antibodies) in the brain homogenate (BH) and post-synaptic density (PSD) of wild type mouse brains. Western blot of 1-month **(A)**, 3-months **(B)**, and 7-months **(C)** brain homogenate and post-synaptic densities show localization of Tpm1.12 in the brain homogenate, but not the post-synaptic compartment throughout the age groups. Total actin and Ponceau staining of blots demonstrates equal loading. **(D)** Western blot analysis of the immunoreactivity for Tpm1.10 and Tpm1.12 in the brain homogenate, synaptic junction and post-synaptic compartment of 3-months-old wild-type mice. **(E)** Due to the poor Tpm1.10 signal in the synaptic junction, the membrane was overexposed to detect Tpm1.10 expression. **(F)** To confirm the specificity of the Tpm1.10 signal in **(E)** the Tm311 antibody, which detects Tpm1.10 but not Tpm1.12 was used, indicating immunoreactivity for Tpm1.10 in the synaptic junction.

Previous immunocytochemical reports, which indicate a pre-synaptic localization of *Tpm1* products, have not discriminated between Tpm1.10 and Tpm1.12 (Had et al., 1994; Guven et al., 2011). This was not possible as the monoclonal α/9c (5-54) antibody recognizes the same antigenic site in exon1b of both Tpm1.10 and Tpm1.12. Our results indicate an absence of Tpm1.10 and Tpm1.12 products from the post-synaptic compartment at 1-, 3-, and 7-months of age (**Figures 3A–C**). Both Tpm1.10 and Tpm1.12 were localized to the synaptic junction fraction, suggesting a pre-synaptic role of these Tpms (**Figures 3D–F**). Given the specificity of the α/9c (5–54) antibody is identical for Tpm1.10 and Tpm1.12, the expression of Tpm1.12 at the pre-synapse was found to be greater than that of Tpm1.10 (**Figure 3E**), with the use of the Tpm1.10 specific antibody Tm311 further confirming the expression of Tpm1.10 in the pre-synaptic compartment (**Figure 3F**).

### Localization of Tpm4.2 to the Post-synaptic Specialization Increases with Age

**FIGURE 4:**
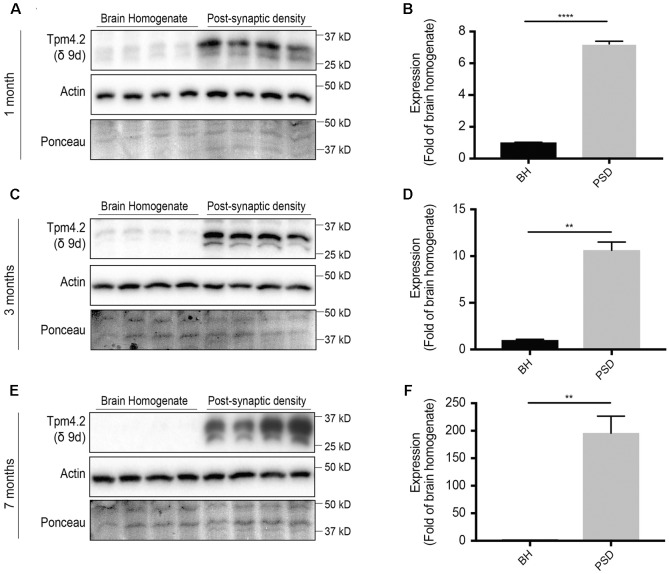
Western blot expression profile of tropomyosin (Tpm) isoform Tpm4.2 (utilizing the δ9d antibody) in the brain homogenate (BH) and post-synaptic density (PSD) of wild type mouse brains. **(A)** Western blot analysis of 1-month-old brain homogenate and PSD fractions. **(B)** Quantification of **(A)** normalized to expression in the brain homogenate. **(C)** Western blots of 3-months-old brain homogenate and PSD fractions. **(D)** Quantification of **(C)** normalized to expression in the brain homogenate. **(E)** Western blot of 7-months-old brain homogenate and PSD fractions. **(F)** Quantification of **(E)** normalized to expression in the brain homogenate. Total actin and Ponceau staining are used as loading controls. (mean ± SEM, *n* = 4, ^∗∗^*P* < 0.01, ^∗∗∗∗^*P* < 0.0001).

Thus far, we have shown that the total pool of Tpm4.2 in the brains of mice decreases with age (**Figures 1C,D**). To determine the comparative levels of Tpm4.2 between the brain homogenate and post-synaptic specialization, equal amounts of protein from either fraction were analyzed via Western blotting, utilizing the δ9d (WD4/9d) antibody (**Figure 4**). We found consistently higher expression in the post-synaptic fraction, compared to levels in the brain homogenate. Furthermore, there was increased recruitment of Tpm4.2 to the post-synaptic compartment with age (**Figures 4A–F**).

### The Expression of Tpm3 Exon 9a-Containing Isoforms Tpm3.3, 5, 8, and 9 Is Reduced at the Post-synaptic Specialization with Age

**FIGURE 5:**
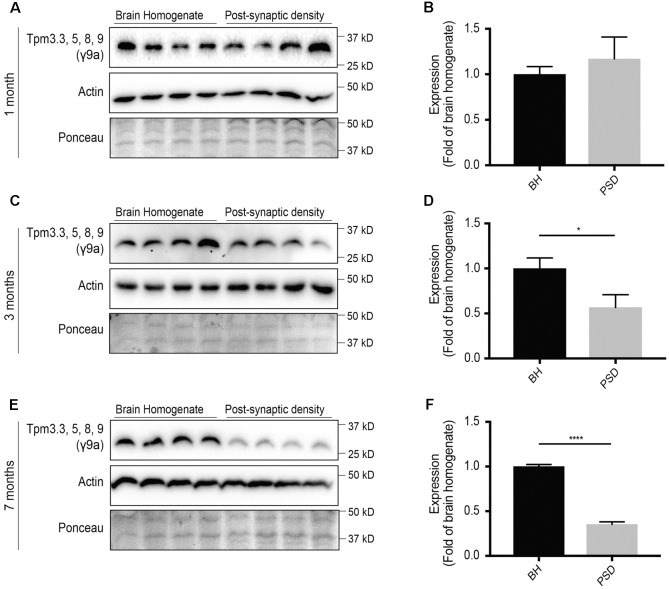
Western blot analysis of expression profile of *Tpm3* exon 9a-containing tropomyosin (Tpm) isoforms (utilizing the γ9a mouse monoclonal antibody) in the brain homogenate (BH) and post-synaptic density (PSD) of wild type mouse brains. **(A)** Western blot of 1-month-old brain homogenate and PSD fractions. **(B)** Quantification of **(A)** normalized to expression in the brain homogenate. **(C)** Western blot of 3-months-old brain homogenate and PSD fractions. **(D)** Quantification of **(C)** normalized to expression in the brain homogenate. **(E)** Western blot of 7-months-old brain homogenate and PSD fractions. **(F)** Quantification of **(E)** normalized to expression in the brain homogenate. Total actin and Ponceau staining are used as loading controls. (mean ± SEM, *n* = 4, ^∗^*P* < 0.05, ^∗∗∗∗^*P* < 0.0001).

The expression of *Tpm3* exon 9a-containing isoforms at the post-synaptic specialization has previously not been analyzed. At 1 month-of-age, there were similar levels of *Tpm3* exon 9a-containing isoforms in the brain homogenate and post-synaptic density fractions (**Figures 5A,B**). By 3 months-of-age, there was a significant reduction in the expression of *Tpm3* exon 9a-containing isoforms in the post-synaptic compartment, compared to levels in the brain homogenate (**Figures 5C,D**), with a further reduction observed at 7 months-of-age (**Figures 5E,F**).

### Expression of Tpm3.4 and Tpm3.7 in the Post-synaptic Fraction Is Limited

**FIGURE 6:**
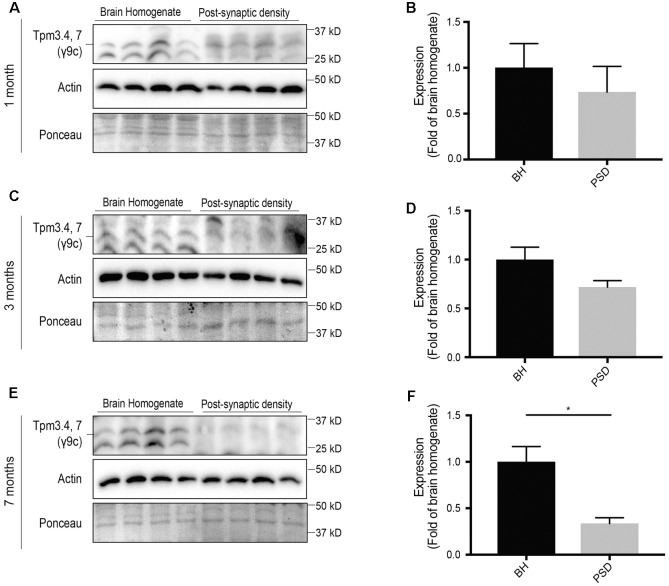
Expression profile of Tpm3 exon 9c-containing tropomyosin (Tpm) isoforms utilizing the γ9c sheep polyclonal antibody in the brain homogenate (BH) and post-synaptic density (PSD) of wild type mouse brains. **(A)** Western blot of 1-month-old brain homogenate and PSD fractions. **(B)** Quantification of **(A)** normalized to expression in the brain homogenate. **(C)** Western blot of 3-months-old brain homogenate and PSD fractions. **(D)** Quantification of **(C)** normalized to expression in the brain homogenate. **(E)** Western blot of 7-months-old brain homogenate and PSD fractions. **(F)** Quantification of **(E)** normalized to expression in the brain homogenate. Total actin and Ponceau staining are used as loading controls. (mean ± SEM, *n* = 4, ^∗^*P* < 0.05).

**FIGURE 7:**
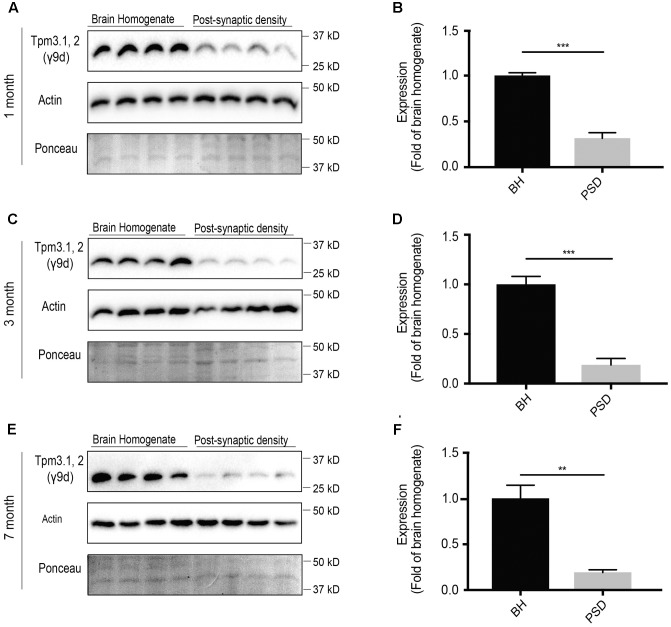
Western blot analysis of expression profile of Tpm3.1 and Tpm3.2 utilizing the polyclonal γ9d sheep antibody in the brain homogenate and post-synaptic density (PSD) of wild type mouse brains. **(A)** Western blots of 1-month-old brain homogenate and PSD fractions. **(B)** Quantification of **(A)** normalized to expression in the brain homogenate. **(C)** Western blots of 3-months-old old brain homogenate and PSD fractions. **(D)** Quantification of **(C)** normalized to expression in the brain homogenate. **(E)** Western blot of 7-months-old brain homogenate and PSD fractions. **(F)** Quantification of (**E**) normalized to expression in the brain homogenate. Total actin and Ponceau staining show equal loading. (mean ± SEM, *n* = 4, ^∗∗^*P* < 0.01, ^∗∗∗^*P* < 0.001).

The Tpm3 exon 9c-containing isoforms Tpm3.4 and Tpm3.7 are brain specific Tpms (Vrhovski et al., 2003). However, their synaptic expression is yet to be characterized. Here, we show that expression of Tpm3.4 and Tpm3.7 in the post-synaptic compartment is limited and decreases with age (**Figure 6**). At 1 month (**Figures 6A,B**), 3 months (**Figures 6C,D**) and 7 months-of-age (**Figures 6E,F**), there was limited immunoreactivity in the post-synaptic fraction when compared to the brain homogenate.

### Tpm3.1 and Tpm3.2 Show Consistent, Albeit Limited, Expression in the Post-synaptic Fraction

Of the *Tpm3* isoforms, the exon 9d-containing isoforms Tpm3.1 and Tpm3.2 are the best characterized. In neuronal cells, Tpm3.1 is involved in modulating axonal length, growth cone size and dendritic branching (Schevzov et al., 2005). In non-neuronal cells, Tpm3.1 is involved in glucose uptake (Kee et al., 2015), maintaining membrane tension (Jalilian et al., 2015) and has been linked to cancer cell transformation (Miyado et al., 1997; Stehn et al., 2013). Therefore, determining the expression of Tpm3.1/2 in the post-synaptic actin cytoskeleton may help shed light on the potential involvement of Tpm3.1/2 in key processes, such as glucose metabolism, in the post-synaptic compartment. At 1-month of age, using the γ/9d sheep polyclonal affinity purified ascites antibody, there was immunoreactivity for Tpm3.1/2 in both the brain homogenate and post-synaptic fraction, with limited immunoreactivity in the post-synaptic fraction when compared to the brain homogenate (**Figures 7A,B**). A similar trend was observed at 3- (**Figures 7C,D**) and 7-months of age (**Figures 7E,F**).

### Tpms Are Differentially Expressed in the Post-synaptic Compartment with Tpm4.2 Being the Most Abundant Tpm

**FIGURE 8:**
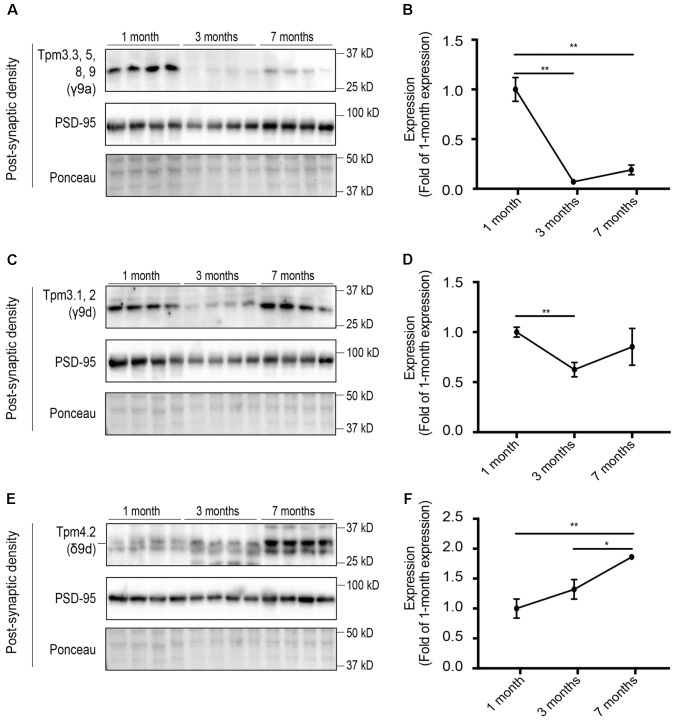
Western blot analysis of the relative expression of tropomyosin (Tpm) isoforms at the post-synaptic density (PSD) in 1, 3, and 7-months-old mice. **(A)** Expression profile of *Tpm3* exon 9a-containing Tpm isoforms utilizing the γ9a mouse monoclonal antibody in the PSD of 1, 3, and 7-months-old mice. **(B)** Quantification of **(A)** normalized to expression at 1-month of age. **(C)** Expression profile of Tpm3.1 and Tpm3.2 utilizing the polyclonal γ/9d sheep antibody in the PSD of 1, 3, and 7-months-old mice. **(D)** Quantification of **(C)** normalized to expression at 1-month of age. **(E)** Expression profile of Tpm4.2, utilizing the δ9d antibody, in the PSD of 1, 3 and 7-months-old mice. **(F)** Quantification of **(E)** normalized to expression at 1-month of age. (mean ± SEM, *n* = 4, ^∗^*P* < 0.05, ^∗∗^*P* < 0.01).

We then analyzed the expression of Tpm isoforms at the post-synaptic specialization, which are suggestive of altered expression with age. Equal amounts of protein from the post-synaptic density fraction of 1-, 3-, and 7-months old mice were analyzed via Western blotting and normalized to expression at 1 month of age (**Figure 8**). *Tpm3* exon 9a-containing isoforms Tpm3.3, 5, 8, 9 were shown to have a high level of immunoreactivity at 1 month of age, with almost negligible expression at 3 and 7 months of age (**Figures 8A,B**). Expression of Tpm3.1/2 in the post-synaptic compartment was found to be relatively similar at 1 and 7 months of age, with decreased expression at 3 months of age (**Figures 8C,D**). Expression of Tpm4.2 in the post-synaptic compartment showed a trend toward increased expression between 1 and 7 months of age (**Figures 8E,F**).

**FIGURE 9:**
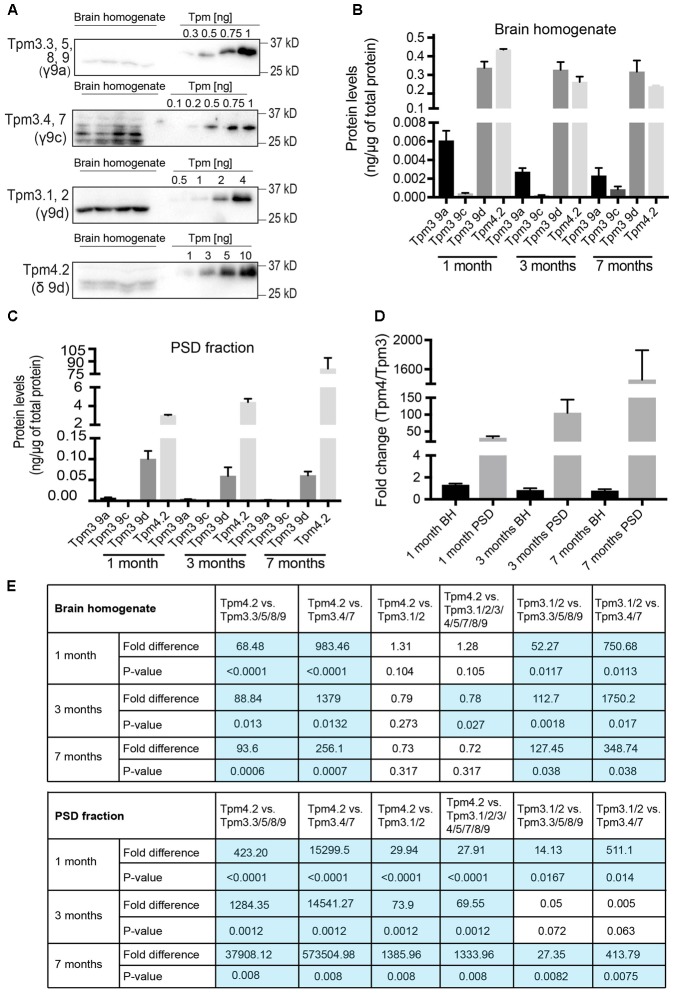
Total protein expression of *Tpm3* and *Tpm4* products. **(A)** Western blot analysis of absolute levels of Tpm expression from *Tpm3* and *Tpm4* in the brain homogenate of 1-month-old mice. Note the higher molecular weight of recombinant Tpm constructs due to a His-tag. **(B)** Quantification of absolute levels of Tpm isoforms from *Tpm3* and *Tpm4* in the brain homogenate of mice. **(C)** Quantification of absolute levels of Tpm isoforms from *Tpm3* and *Tpm4* in the post-synaptic density of mice. **(D)** Comparative levels of the contribution of Tpm3 versus Tpm4 isoforms to the total output from *Tpm3* and *Tpm4* in brain homogenate (BH) and the post-synaptic density (PSD) fraction. **(E)** Fold difference in expression of Tpm4.2 compared to Tpm3 isoforms in brain homogenate. (I) Fold difference in expression of Tpm4.2 compared to Tpm3 isoforms in the post-synaptic density fraction. Shown are *p*-values comparing individual Tpm isoforms. (mean ± SEM, *n* = 4). Significant differences in **(E)** are highlighted in blue.

To determine the absolute contribution of individual Tpm isoforms from *Tpm3* and *Tpm4* to the total pool of Tpms from these two genes, brain tissue samples were compared to defined concentrations of recombinant Tpm proteins (**Figure 9A**). The defined concentrations of recombinant proteins (**Figure 9A**) were used to generate standard curves, from which the concentrations of Tpms in the brain homogenate sample were determined (ng of Tpm protein expressed per μg of total cellular protein). The amount of Tpm proteins in the brain homogenate could then be compared to the amounts in the PSD fraction. Absolute expression levels of Tpm 3.1/2, Tpm3.3/5/8/9, Tpm 3.4/7 and Tpm 4.2 were compared with each other in total brain homogenate (**Figure 9B**) and PSD fractions (**Figure 9C**). The ratio of Tpm4.2 over the sum of all Tpm3 isoforms (**Figure 9D**) identifies Tpm4.2 as the major post-synaptic Tpm, expressed in mouse brain tissue. Whereas Tpm4.2 expression was comparable to Tpm3.1/2 expression in the whole brain tissue, Tpm4.2 showed an approximately ∼30-fold, ∼74-fold, and ∼1386-fold higher expression compared to the highest expressed Tpm3 isoforms Tpm3.1/2 at 1, 3 and 7 months, respectively (**Figures 9D,E**).

## Discussion

Although the actin cytoskeleton plays an integral role at the synapse, and Tpms are known to generate distinct F-actin populations (Gunning et al., 2008; Gateva et al., 2017), there is limited characterization of the Tpm expression profile at the post-synaptic compartment. This study is the first to identify an age-dependent targeting of Tpm isoforms to the post-synaptic compartment. Via the biochemical fractionation of PSDs and the adherent sub-synaptic web material, we identified the age-related expression of Tpm4.2 in the post-synaptic compartment in the mouse brain. Although other Tpm isoforms from *Tpm3* were expressed in the PSD fraction, none show such a consistent presence as Tpm4.2, particularly in comparison to total expression levels in the brain.

Previous studies have shown that *Tpm1* products are expressed in the pre-synaptic compartment (Had et al., 1994; Weinberger et al., 1996; Vrhovski et al., 2003; Guven et al., 2011). However, the biochemical approach used in this study, has also allowed us to confirm that both Tpm1.10 and Tpm1.12 are absent from the post-synaptic compartment *in vivo* as well as highlighting the relative difference in expression levels between Tpm1.10 and Tpm1.12. Although Tpm1.12 is more highly expressed than Tpm1.10, both isoforms follow an almost identical expression profile from early development through to older mice. This suggests that Tpm1.12, rather than Tpm1.10, may have a more dominant functional role in the pre-synaptic compartment. Mechanistically, Tpm1.12 but not Tpm1.10 has been shown to increase cell stiffness (Jalilian et al., 2015). Tpm1.12 has also been associated with the formation of shorter, less stiff F-actin via the recruitment and binding of cofilins to F-actin (Bryce et al., 2003). Together, this suggests that Tpm1.12 may be a ‘key regulator’ of pre-synaptic actin dynamics, potentially impacting on the stiffness of the pre-synaptic membrane and influencing the structure of pre-synaptic F-actin.

In the central nervous system, alternative splicing of *Tpm3* gives rise to the expression of 9 isoforms (Dufour et al., 1998; Vrhovski et al., 2003). Therefore, determining the isoform-specific localization, expression and function of these isoforms is a complex task. Overall, we have shown a decrease in Tpm3 expression with aging. Previous studies indicated that total Tpm3 expression in the brain is constant (Vrhovski et al., 2003). However, in this study the authors did not state the precise age of the mice at ‘adulthood.’ Therefore, it may be possible that they did not investigate Tpm3 expression up to the same age as in our current study. Further evidence for the significant role of *Tpm3* products in embryonic development and cell survival can be seen in genetic deletion studies. Unlike *Tpm4* knockout (Pleines et al., 2017), *Tpm3* knockout is embryonically lethal (Hook et al., 2004). Fifty-percent of mice with deletion of two alleles from *Tpm3* isoforms fail to survive embryogenesis (Hook et al., 2004). Together, this suggests a role for *Tpm3* products in development and early adulthood, with decreased expression in older mice. Specifically, the high level of *Tpm3* exon 9a-containing isoform expression at 1 month of age in the brain homogenate and PSD fraction indicates that one or more of the Tpm isoforms, arising from this exon, may be important regulators of F-actin during adolescence. This is supported by previous proteomic studies, which show an increase in protein expression, involved in neurite outgrowth and metabolism of ATP, early in development (McClatchy et al., 2012). For the first time, we can also show that the contribution of *Tpm3* exon 9c-containing Tpms in the post-synaptic compartment appears to be minimal. Given the vast role of Tpm3.1 in neuronal function and signaling pathways and the detection of *Tpm3* products in the post-synaptic compartment (Guven et al., 2011), it was anticipated that Tpm3.1 would also play a prominent role at the post-synaptic compartment. The data presented thus far has highlighted that although Tpm3.1/2 are present in the PSD fraction, they do not appear enriched and, therefore, may not have a key role in modulating F-actin dynamics at the post-synaptic specialization.

Tpm4.2 has long been associated with the post-synaptic compartment (Had et al., 1994; Guven et al., 2011). In this study, we have further identified an age-dependent targeting of Tpm4.2 to the post-synaptic compartment, with a concomitant decrease in the expression levels of Tpm4.2 in the brain homogenate. This suggests that there is a direct targeting of Tpm4.2 to different F-actin populations throughout development, with a specific targeting to the PSD in older mice. How the specific targeting of Tpm4.2 to the post-synaptic specialization occurs, and changes over time, remains unknown and needs further investigations. One mechanism could be via the interaction between Tpm4.2 and formins. Formins are a large family of proteins known to initiate the nucleation of new actin filaments and modulate the rate of elongation at the barbed end of actin filaments (Li and Higgs, 2003). It has been proposed that the Tpm composition of F-actin is organized by formins at the time of nucleation (Johnson et al., 2014; Gunning et al., 2015b). This model is supported by *in vivo* work in yeast, which shows that switching the location of specific formins directly alters the Tpm associated with the actin polymer. Furthermore, this also redirects the location of myosin motors within the cell (Johnson et al., 2014). Tpm4.2 has been directly implicated in this interaction between formins, Tpms and myosin motors (Tojkander et al., 2011). Tojkander and colleagues showed that in the absence of Tpm4.2, myosin II was not recruited to stress fibers in osteosarcoma cells. Additionally, in the absence of Tpm4.2, the actin filaments nucleated by the formin Dia2 preferentially formed filopodia rather than become precursors of transverse arcs. In cultured mouse neurons, mDia2 expression increases during the development of dendritic spines (Hotulainen et al., 2009), supporting its role in the reorganization of the actin cytoskeleton during synapse formation. Furthermore, partial silencing of mDia2 function via RNAi causes a decrease in the number of immature type spines and an increase in more mature, stubby shaped spines as well as an increase in the mean dendritic spine head width (Hotulainen et al., 2009). Another formin that is also involved in spine formation and maintenance is FMN2. The inactivation of mouse *Fmn2* causes a 32% reduction in the number of spines in mice (Law et al., 2014) indicating that multiple formin isoforms are needed for proper spine formation and maintenance. It remains to be seen whether Tpm4.2 is targeted to the post-synaptic compartment in a formin-dependent manner and if there is preferential targeting of Tpm4.2 by a specific formin isoform. If this interaction between formins and Tpm4.2 is found to be true, it could also help explain the recruitment of the myosin II motor protein into the compartment. Interestingly, a recent proteomic study of the human neocortex identified Tpm4.2 in the post-synaptic compartment and classified it in a proteome cluster along with MAGUK scaffolding proteins and the KEGG pathway term glutamatergic synapse (Roy et al., 2017). Together this suggests that, also in humans, Tpm4.2 is associating with the scaffolding proteins, directly associated with the PSD and may play a role in the organization of the postsynaptic scaffold and thereby influencing the function of glutamatergic synapses.

By analyzing the PSDs fractionated from whole mouse brain tissue, region-specific effects may have been masked. Although post-synaptic cytoskeletal proteins have been shown to have the lowest variability between regions of the neocortex (Roy et al., 2017), compartment-specific and age-specific localization of synaptic proteins has been documented. For example, while AMPA receptor subunits are enriched in the PSD of cortical and cerebellar tissue, hippocampal tissue is associated with elevated extra-synaptic AMPA receptor subunits (Pandya et al., 2017). Likewise, developmental proteomic studies of synaptosomes indicate enrichment of proteins, associated with neurite outgrowth, early in development and enrichment of proteins, associated with synaptic transmission, later in development (McClatchy et al., 2012). Together with our data, this suggests that Tpm4.2 may be associated with actin-dependent regulation of synaptic transmission. Furthermore, differences in protein expression between brain regions, as analyzed by synaptosomal proteomics, have also been identified to be the greatest in adult rats (McClatchy et al., 2012), indicating the need to analyze the expression of Tpm4.2 in different brain regions of adult mice. Therefore, the analysis of adult and aged Tpm4.2 knockout mice (Pleines et al., 2017) for changes in synaptic structure and transmission and behavioral testing may further elucidate the role of Tpm4.2 in synaptic structure and function.

With the exception of drebrin, where there is a developmental shift from drebrin E to drebrin A during synapse maturation (Kojima et al., 2016), little is documented on how localization of actin associated proteins, such as Tpms, to the post-synaptic specialization changes throughout development. The temporal changes in the diversity of Tpm expression at the post-synaptic specialization, identified here, are likely to play a role in the maturation and function of synaptic connections between neurons. We have shown that there is differential expression of Tpms at the post-synaptic compartment, with Tpm4.2 being highly expressed in older mice. Therefore, by taking advantage of the distinct localization pattern of Tpm4.2 to the post-synaptic compartment, we can potentially manipulate the post-synaptic actin cytoskeleton via pharmacological means in neurodegenerative disorders. Pharmacological means for targeting specific Tpm isoforms are currently being explored for other, non-neuronal diseases, such as cancer (Stehn et al., 2013; Currier et al., 2017).

## Author Contributions

TF supervised the project. TF and AS designed the research, performed the research and analyzed the data. AS and TF wrote the paper. PG and EH provided materials. JP, PG, and EH helped editing the manuscript. All authors read and approved the final manuscript.

## Conflict of Interest Statement

The authors declare that the research was conducted in the absence of any commercial or financial relationships that could be construed as a potential conflict of interest.
