# Supplementary figures and images for: Developmental Profiling of Tropomyosin Expression in Mouse Brain Reveals Tpm4.2 as the Major Post-synaptic Tropomyosin in the Mature Brain
Source: Front Cell Neurosci. 2017 Dec 22;11:421. doi: 10.3389/fncel.2017.00421 (PMC5743921; doi:10.3389/fncel.2017.00421)

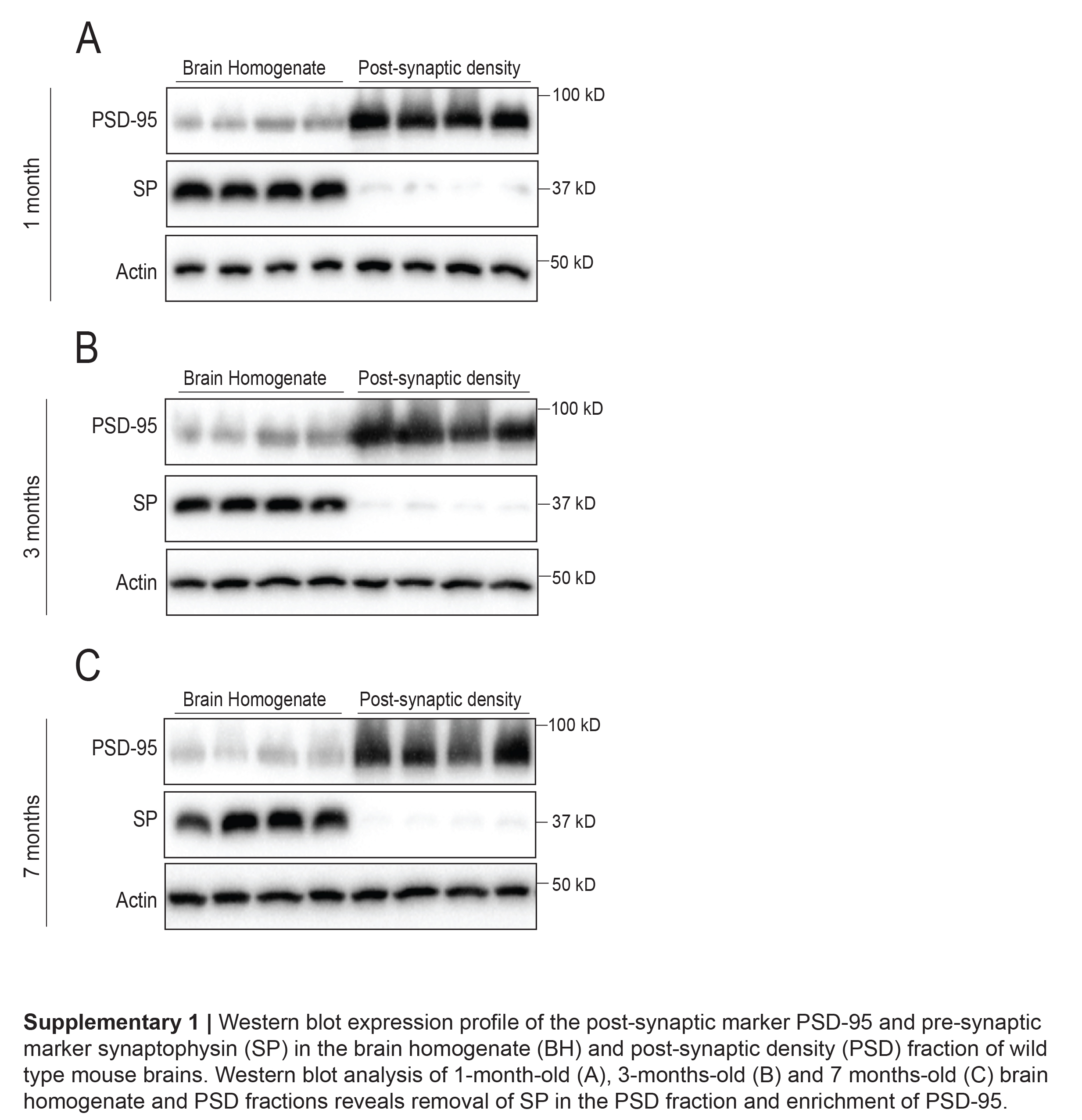

Supplement: Supplementary file 1 [file Image_1.JPEG]
